# Laboratory-Based Surveillance and Genetic Diversity of Enteric Adenovirus Among Children in Argentina, 2022–2024

**DOI:** 10.3390/tropicalmed11020035

**Published:** 2026-01-25

**Authors:** Juan Ignacio Degiuseppe, Paula Mabel Moron, Christian Barrios Mathieur, Argentinean Surveillance Network for Viral Gastroenteritis

**Affiliations:** Laboratory of Viral Gastroenteritis, National Institute for Infectious Diseases (INEI)-ANLIS “Dr. Carlos G. Malbrán”, Ciudad Autónoma de Buenos Aires, Buenos Aires 1281, Argentina; pmoron@anlis.gob.ar (P.M.M.); cbarrios@anlis.gob.ar (C.B.M.)

**Keywords:** enteric adenovirus, pediatric gastroenteritis, Argentina

## Abstract

Enteric adenoviruses are recognized causes of pediatric acute gastroenteritis, yet national-level data on their epidemiology and genetic diversity in Argentina remain limited. This study aimed to describe the laboratory-based surveillance of enteric adenoviruses and to characterize the circulating genotypes among children during the 2022–2024 period. Data were obtained from the Argentine National Health Surveillance System, including weekly aggregated reports of adenovirus testing results from clinical laboratories nationwide. Detection frequencies were analyzed by year, age group, clinical setting, geographic region, and epidemiological week. Molecular characterization was performed using partial hexon gene sequencing. A total of 22,826 stool samples were tested, of which 1530 (6.7%) were positive for adenovirus, with no significant differences in positivity across years. Detection rates were highest among children under 1 year of age and were consistently greater in outpatient and emergency department settings compared with hospitalized patients. No clear seasonal pattern was observed. Genotyping revealed a predominance of HAdV-F41, with sporadic detection of non-enteric adenovirus types. These findings provide the first nationwide overview of enteric adenovirus circulation and genetic diversity in Argentina, highlighting stable transmission patterns and supporting the value of sustained laboratory surveillance to better characterize viral gastroenteritis etiology in the post-rotavirus vaccination era.

## 1. Introduction

Human adenoviruses (HAdVs) are globally distributed pathogens that cause a wide spectrum of clinical manifestations. Although they can be associated with various syndromes, their greatest impact occurs in respiratory and gastrointestinal diseases, particularly among young children [1]. HAdVs show a marked predilection for the pediatric population under five years of age, who often spend prolonged periods in closed environments such as daycare centers, schools, and other institutional settings, favoring person-to-person transmission. This age group is therefore a key target for viral surveillance, as it represents an important population in terms of both disease burden and viral spread [2].

HAdVs belong to the genus Mastadenovirus and are classified into seven species (A–G) based on biological, biochemical, and genomic properties, including the guanine-plus-cytosine content of their DNA [3]. To date, 116 distinct HAdV types have been identified [4]. While types up to 51 were defined by serological criteria, more recent types are classified using genotypic approaches, based on the identification of novel sequences or recombinant phylogenies involving major capsid genes (hexon, penton, and fiber) [5]. Among these species, HAdV-F (commonly referred to as enteric adenoviruses and comprising types HAdV-F40 and HAdV-F41) shows a marked tropism for the gastrointestinal tract and is primarily associated with acute gastroenteritis [6,7]. Although this association has long been recognized, enteric adenoviruses have recently regained attention after being proposed as potential etiologic agents of severe acute hepatitis of unknown origin, reported mainly in the United States and Europe [8].

In Argentina, acute non-bloody diarrheal diseases of all causes are reported weekly to the National Health Surveillance System (SNVS2.0), stratified by age group. In contrast, laboratory-based surveillance provides greater specificity by distinguishing between parasitic, bacterial, and viral etiologies. Among viral causes, events associated with rotavirus, enteric adenovirus, and—more recently—norovirus are routinely notified [9].

Despite the recognized relevance of viral gastroenteritis, few studies have addressed the epidemiology and molecular diversity of enteric adenoviruses in Argentina, and available data are generally outdated or geographically limited. A recent investigation showed that, after rotavirus and norovirus, enteric adenoviruses were the third most frequently detected viruses in non-inflammatory diarrheal disease [10].

Therefore, the aim of this study was to analyze laboratory-based surveillance data reported to the SNVS2.0 on enteric adenovirus–associated acute gastroenteritis, as well as to describe the genetic diversity of circulating HAdV types in Argentina during the 2022–2024 period among children.

## 2. Materials and Methods

Throughout this study, the term *enteric adenovirus detection* is used in an operational sense and refers to adenovirus antigen detection in stool samples obtained within the context of viral gastroenteritis surveillance, as determined by immunochromatographic assays. Data were obtained from the Viral Diarrhea Notification module of the SNVS2.0. In summary, clinical laboratories across the country submit weekly aggregated reports indicating the total number of tests performed for enteric adenoviruses and the number of positive results, all of which are generated using immunochromatographic assays that do not discriminate between enteric and non-enteric adenovirus types. Consequently, the number of tests performed in each reporting laboratory reflects the actual diagnostic demand from local physicians. Reported data are organized by patient age group, clinical setting (outpatient/emergency department or inpatient), and epidemiological week (EW). The epidemiological week (EW) is an internationally standardized time unit used for disease surveillance, allowing temporal trends to be compared consistently across years. Each EW begins on Sunday and ends on Saturday, with 52 weeks defined per calendar year. To maintain consistent representativeness of the temporal series and to ensure continuity in laboratory reporting, only data from laboratories that reported results for at least 44 epidemiological weeks per year (approximately 85%) to the SNVS2.0 were included in the analysis. This threshold was applied as a data quality criterion to ensure sustained reporting over time and to minimize potential bias related to intermittent notification, particularly in analyses of weekly and seasonal patterns.

For the present analysis, we estimated the annual and global detection frequency, seasonal distribution, and age group patterns for the 2022–2024 period. Data were also stratified by province and subsequently grouped into three geographic regions (North, Center, and South). Comparative analyses were performed to evaluate differences in enteric adenovirus detection rates across years, age groups, clinical settings, and geographic regions. Proportions were compared using contingency tables, and statistical significance was assessed with the chi-square test when appropriate. For analyses involving repeated comparisons of aggregated indicators across time or regions (e.g., regional detection rates compared across different years), non-parametric methods were applied to account for the matched structure of the data and potential deviations from normality. In these cases, post hoc pairwise comparisons were conducted using the Wilcoxon signed-rank test with adjustment for multiple testing. Inferential analyses were used to complement descriptive comparisons within the context of routine, passive surveillance, rather than to model temporal trends or causal associations. All statistical analyses were performed using InfoStat software, version 2020p (https://www.infostat.com.ar/index.php?mod=page&id=46, accesed on 14 November 2025), and statistical significance was defined as *p* < 0.05. Very small *p*-values are reported as *p* < 0.0001 when applicable.

Molecular characterization of circulating adenovirus strains was performed on all adenovirus-positive stool specimens that were referred to the National Reference Laboratory within the framework of routine enteric virus surveillance activities. Sample referral represents a subset of reported positive cases and is based on logistical feasibility, specimen availability, and local laboratory capacity, with the objective of preserving representativeness across calendar years, geographic regions, and clinical settings. Genotyping was conducted by partial sequencing of the hexon gene, a major capsid protein commonly used for molecular typing [11,12]. Quality control criteria were applied prior to genotype assignment. Sequence quality was evaluated based on amplicon coverage and bidirectional consistency. When initial sequencing results did not meet these criteria, amplification and sequencing were repeated whenever sufficient specimen material was available. Sequences were considered acceptable for genotyping when they covered at least 90% of the expected amplicon length and showed concordant forward and reverse reads during consensus sequence generation. Samples for which no amplicon could be obtained after repeated attempts were classified as non-typeable. Obtained sequences were analyzed using the BLAST+ 2.17.0 algorithm [13] to determine genotype assignment based on nucleotide identity with reference sequences available in GenBank. For each sequence, the top ten BLAST matches corresponding to distinct submissions were evaluated, ensuring consistent genotype assignment and minimizing redundancy.

## 3. Results

### 3.1. Global Enteric HAdV Detection

During the study period, 69, 86, and 74 laboratories reported data to the SNVS2.0 in 2022, 2023, and 2024, respectively. Overall, 22,826 stool samples from children younger than 10 years of age were tested for enteric adenoviruses, of which 1530 (6.7%) yielded positive results. The annual detection rate showed only moderate variation over time, with the highest proportion observed in 2023 (7.8%), followed by 2022 (6.8%) and 2024 (5.6%). The total number of samples analyzed increased steadily across the study period, rising from 6788 in 2022 to 8527 in 2024. Although a slight decline in positivity was observed in 2024 compared with the two preceding years, the differences across years were modest and did not reach statistical significance (*p* = 0.052). These findings indicate a relatively stable circulation of enteric adenoviruses during the three-year surveillance period (Table 1).

### 3.2. Age-Specific Distribution of Enteric HAdV Detection

When analyzed by age group, enteric adenovirus detection was highest among infants younger than 1 year (8.2%) and children aged 1 year (7.7%), whereas lower positivity rates were observed in older children, including those aged 2–4 years (5.7%) and 5–9 years (5.0%). Over the entire study period, infants younger than 1 year represented the largest proportion of tested samples (28.7%), followed by children aged 2–4 years (27.9%) and those aged 1 year (22.5%). Across individual years, the highest positivity rates consistently occurred in the youngest age groups (<1 year and 1 year), particularly in 2023, when detection reached 9.6% and 9.0%, respectively. In contrast, children aged 5–9 years showed the lowest detection frequencies throughout the study period. Overall, an age-related gradient was noted, with positivity rates declining progressively with increasing age. The overall comparison among age groups revealed a statistically significant difference (*p* < 0.0001), indicating that younger children were more likely to test positive for enteric adenovirus (Table 1).

### 3.3. HAdV Detection by Clinical Setting

During the 2022–2024 period, a total of 22,826 stool samples from children younger than 10 years of age were tested for enteric adenoviruses. Of these, 36.4% corresponded to outpatient or emergency department encounters, whereas 63.6% were obtained from hospitalized patients. The overall detection rate was significantly higher among outpatient samples (9.7%) than among inpatient samples (5.0%) (*p* < 0.0001). When analyzed by year, this difference was consistently observed across the study period. In 2022, positivity reached 11.0% among outpatients compared with 4.4% among inpatients (*p* < 0.0001). Similarly, in 2023, detection rates were 10.2% and 6.4%, respectively (*p* < 0.0001), while in 2024, positivity declined to 8.0% among outpatients and 4.3% among inpatients (*p* < 0.0001). The relative frequency proportion of reported tests by clinical setting remained stable over time, with hospitalized patients accounting for approximately two-thirds of all tested samples (ranging from 62.0% to 65.1%) and outpatient or emergency department cases contributing roughly one-third (ranging from 34.9% to 38.0%) (Table 2).

### 3.4. Detection of HAdV by Geographic Region

Positivity rates for enteric adenoviruses differed significantly across geographic regions over the study period (*p* < 0.0001). The Southern region consistently showed the highest detection rates, with values of 14.0% in 2022, 15.8% in 2023, and 8.8% in 2024 (overall mean: 12.8%). The Central region exhibited intermediate positivity levels (7.0%, 6.4%, and 5.7%; overall mean: 6.3%), whereas the Northern region presented the lowest rates (3.2%, 9.7%, and 3.6%; overall mean: 5.5%). Pairwise comparisons confirmed that positivity in the Southern region was significantly higher than in both the Central and Northern regions (*p* < 0.0001 for both comparisons). In contrast, no statistically significant difference was observed between the Central and Northern regions (*p* = 0.14). When temporal variation was assessed, overall positivity rates showed only moderate fluctuations across years (6.8% in 2022, 7.8% in 2023, and 5.6% in 2024), with no statistically significant year-to-year differences (*p* = 0.12). The relative frequency proportion of reported tests by region remained stable throughout the study period (*p* = 0.65). The Central region accounted for approximately three-quarters of all determinations (mean %RFP: 74.1%), followed by the Northern (17.6%) and Southern (8.3%) regions (Table 3).

### 3.5. Seasonal Distribution of Enteric HAdV Detections

**Figure 1:**
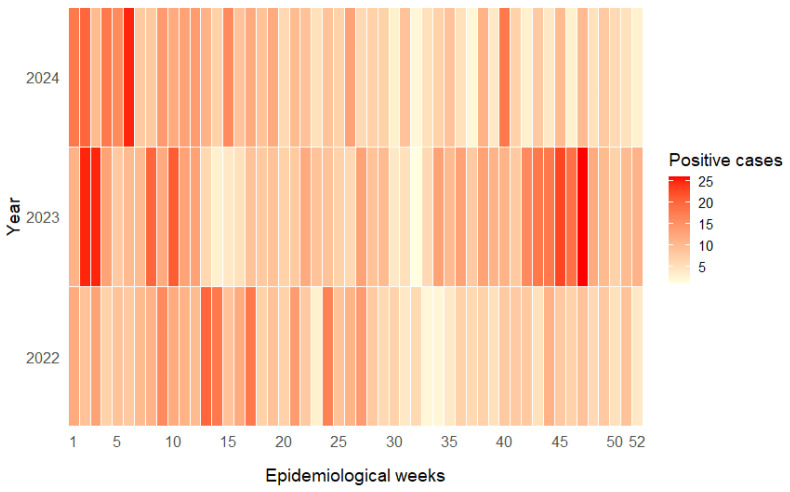
Weekly distribution of enteric adenovirus–positive cases by epidemiological week, Argentina, 2022–2024. Heatmap showing the absolute number of enteric adenovirus–positive stool samples reported in Argentina during 2022, 2023, and 2024. Epidemiological weeks (EW) range from EW1 (first week of January) to EW52 (last week of December). A color code is used to represent the number of positive cases reported per epidemiological week, with darker shades indicating higher counts.

Analysis of weekly enteric adenovirus detections did not reveal a consistent or clearly defined seasonal pattern across the three study years (Figure 1). In 2022, cases were reported throughout the entire year, with only mild increases observed during late summer to early autumn (EWs 8–14) and again in early winter (EWs 23–27). In 2023, the distribution was more heterogeneous, showing a broader elevation in detections from late autumn to early winter (EWs 20–30), along with smaller secondary increases toward the end of the year. During 2024, case numbers remained relatively stable across epidemiological weeks, with only minor fluctuations and no clear temporal concentration. When data from the three years were combined, a weak but statistically significant deviation from a uniform weekly distribution was observed (*p* = 0.002), indicating the presence of minor temporal fluctuations rather than sustained clustering. These variations included slightly higher detection during late autumn and early winter (approximately May–July in the Southern Hemisphere). However, the overall temporal dispersion index was close to unity (variance-to-mean ratio = 1.12), suggesting an approximately random distribution of cases over time. Taken together, these findings do not support the presence of a marked seasonal pattern and instead indicate continuous or weakly seasonal circulation of enteric HAdV in Argentina, characterized by multiple low-amplitude peaks distributed throughout the year.

### 3.6. Genetic Diversity of HAdV-Positive Stool Samples

**Figure 2:**
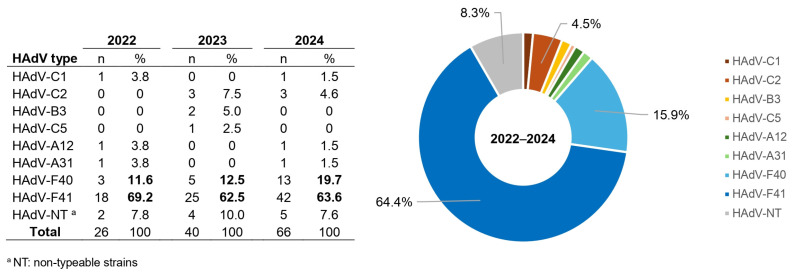
Distribution of adenovirus types detected in Argentina, 2022–2024. Distribution of adenovirus types identified during the study period in Argentina. The figure includes a table summarizing the annual distribution of detected adenovirus types for 2022, 2023, and 2024, and a ring (donut) chart showing the overall distribution for the entire 2022–2024 period. Adenovirus types are displayed using a color code. In the table, bold formatting highlights the two most frequently detected adenovirus types for each year. In the ring chart, percentage labels are shown only for adenovirus types with a relative detection frequency greater than 4% to improve readability.

Between 2022 and 2024, a total of 1530 enteric adenovirus–positive stool samples were reported nationwide through the surveillance network. Of these, 132 samples (8.6%) were referred to the National Reference Laboratory and successfully genotyped. Sample referral for molecular characterization was based on logistical feasibility, specimen availability, and local laboratory capacity, rather than on clinical or epidemiological characteristics. Within these constraints, efforts were made to ensure representation across years, geographic regions, and clinical settings. Nine distinct HAdV types were identified, corresponding to species A (types 12 and 31), B (type 3), C (types 1, 2, and 5), and F (types 40 and 41). Overall, HAdV-F41 was the predominant genotype, accounting for 64.4% (85/132) of all typed strains, followed by HAdV-F40, which represented 15.9% (21/132). Non-typeable (NT) strains accounted for 8.3% (11/132) of the total, whereas all other detected types were identified only sporadically, each at frequencies ≤4.5%. When stratified by year, HAdV-F41 consistently remained the most frequently detected genotype throughout the study period, representing 69.2% of typed strains in 2022, 62.5% in 2023, and 63.6% in 2024. The proportion of HAdV-F40 increased modestly over time, rising from 11.5% in 2022 to 19.7% in 2024. In contrast, the proportion of NT strains remained low and relatively stable across years, ranging from 7.7% to 10.0%. Other HAdV types, including HAdV-C1, C2, C5, A12, A31, and B3, were detected sporadically and without a consistent temporal pattern. Overall, no statistically significant differences were observed in the relative frequencies of HAdV-F41 and HAdV-F40 across the three study years (*p* = 0.37), indicating a stable pattern of circulating enteric adenovirus genotypes during the study period (Figure 2).

## 4. Discussion

Beyond its national scope, this study provides a real-world characterization of enteric adenovirus detection within a routine, laboratory-based surveillance system. By relying on data generated through standard diagnostic and reporting practices, these findings contribute to understanding how enteric adenoviruses are detected, reported, and monitored in a middle-income country setting, and offer a baseline for future comparisons and surveillance-strengthening efforts. This work represents the first nationwide analysis of enteric adenovirus surveillance among children in Argentina, integrating epidemiological and molecular information to describe detection trends, clinical settings, regional distribution, and genotype diversity over a three-year period. The results should be interpreted in the context of long-standing rotavirus vaccination programs and the post–COVID-19 period, both of which may have influenced healthcare utilization and diagnostic practices, rather than as evidence of direct changes in adenovirus epidemiology. Together, these observations provide updated epidemiological context for viral gastroenteritis surveillance in pediatric populations. The progressive increase in the number of analyzed samples over time likely reflects both the expansion of participating laboratories within the national surveillance network and increased diagnostic awareness among clinicians. This expansion may also be influenced by heightened interest in adenovirus following reports of acute hepatitis of unknown origin and by the broader implementation of molecular diagnostic platforms in recent years. Taken together, these developments may have improved the sensitivity of the surveillance system, allowing a more comprehensive description of adenovirus circulation and its epidemiological behavior in Argentina.

The annual positivity rates for enteric adenoviruses exhibited only moderate variation during the 2022–2024 period, supporting the interpretation of relatively stable circulation over time. The overall proportion of enteric adenovirus detection observed in this study was lower than that reported for South America in a recent systematic review and meta-analysis [14], but was closer to estimates from a recent global analysis reporting an overall pooled prevalence of approximately 6.0% [15]. Within the national context, and considering the diagnostic approach used in this surveillance strategy, the observed positivity rates were similar to those reported in Egypt (~7%) and Lebanon (~6%) [16,17], while remaining lower than estimates from Iraq (~17%) and India (~10%) [18,19], and higher than those documented in Nepal and Japan (~3%) [20,21]. These differences likely reflect a combination of true epidemiological variability and heterogeneity in study populations, diagnostic methods, and surveillance intensity, rather than substantial differences in viral circulation alone.

No clear temporal pattern was observed, suggesting that enteric adenovirus circulation occurs throughout the year with only moderate fluctuations. This epidemiological behavior contrasts with that of other viral gastroenteritis agents, such as rotavirus and norovirus, which typically display well-defined winter peaks in temperate regions [22]. When analyzed by age group, detection rates were consistently higher among children younger than two years and declined progressively with increasing age. This pattern is consistent with previous evidence indicating that adenovirus-associated gastroenteritis predominantly affects early childhood, when susceptibility to enteric pathogens is higher and protective immunity has not yet fully developed [15,23]. Nevertheless, this age-related gradient should be interpreted cautiously, as differences in healthcare-seeking behavior and diagnostic practices across age groups may also contribute to the observed pattern. Younger children are more likely to receive medical attention and to undergo etiological testing for diarrheal illness, whereas milder cases in older children may be underrepresented in surveillance data. Accordingly, the observed differences in positivity between age groups likely reflect both biological susceptibility and surveillance-related factors, rather than an independent effect of age alone.

The higher proportion of enteric adenovirus–positive samples observed among outpatient and emergency department cases should likewise be interpreted within the context of passive, laboratory-based surveillance. Although this pattern could be compatible with a predominance of mild or moderate gastroenteritis episodes, it more likely reflects differences in healthcare-seeking behavior and diagnostic practices across clinical settings. In outpatient contexts, laboratory testing is often requested selectively, typically for patients presenting with more severe symptoms or when etiological confirmation is considered necessary. This selective testing may lead to an overrepresentation of positive results. Consequently, differences in testing criteria and access to diagnostic resources, rather than differences in disease severity, may partly explain the observed distribution of positivity across settings.

Regional variations in enteric adenovirus detection rates within Argentina were moderate and did not reveal a consistent geographic pattern. The Central region contributed the largest proportion of tests nationwide, a finding that can be explained by its higher population density, concentration of urban centers, and the presence of most tertiary-care hospitals and laboratories with greater diagnostic capacity. In contrast, regions with more limited economic resources and greater rural dispersion rely more heavily on clinical diagnosis rather than etiological confirmation, resulting in lower testing volumes and potential underrepresentation in surveillance data. Despite these quantitative differences, variation in positivity rates across regions did not follow a clear geographic gradient. Notably, the highest detection rates were recorded in the South, while the North—previously identified as a high-burden area for other enteric viruses [9,24]—showed lower proportions of adenovirus positivity. These findings suggest that contextual and structural factors, including differences in healthcare infrastructure, surveillance intensity, and potentially climatic conditions, may influence the observed regional variability. Further investigations will be required to better elucidate the contribution of these factors to adenovirus circulation dynamics in Argentina.

The genetic characterization of enteric adenoviruses showed that approximately three-quarters of the successfully genotyped samples corresponded to HAdV-F41, confirming its predominance as the main type associated with acute gastroenteritis in children [14,15,25,26]. Other genotypes belonging to species A and C were detected sporadically, in line with occasional reports from previous studies. These observations highlight the need for broader clinical and molecular investigations to better understand the potential pathogenic role of non-F adenoviruses in gastrointestinal infections. Although genotyping was performed using partial hexon gene sequencing and BLAST-based identification, two samples collected in 2024 and initially classified as HAdV-C2 showed close similarity to the recently described HAdV-C89 type. Because additional genomic regions were not analyzed, recombination could not be formally confirmed, and these findings should therefore be interpreted cautiously. This limitation underscores the constraints of hexon-only typing for identifying recombinant adenoviruses [27]. Accordingly, current recommendations emphasize the inclusion of penton base, hexon, and fiber genes—or whole-genome sequencing—to more accurately characterize adenovirus evolutionary dynamics, particularly within species B and C [27,28,29].

Several limitations should be considered when interpreting these findings. All participating laboratories relied on immunochromatographic assays for adenovirus detection, which represent the most widely used diagnostic approach in routine clinical practice and surveillance systems in middle-income and resource-limited settings. Although these assays enable broad and timely case detection at the national level, their sensitivity and specificity may vary across commercially available tests when compared with molecular methods. As a result, surveillance estimates may be influenced by false-positive results or by the detection of non-enteric adenovirus species shed in feces. Therefore, adenovirus detection in stool samples should be interpreted with caution, as immunochromatographic assays do not discriminate between enteric and non-enteric adenovirus types. The identification of non-enteric adenoviruses does not necessarily indicate a causal role in gastroenteritis, but may instead reflect intestinal excretion following respiratory or systemic infections, as illustrated by the occasional detection of HAdV-B3, a type primarily associated with respiratory disease. This approach reflects routine surveillance practices and the operational constraints inherent to passive laboratory-based systems. In addition, a small proportion of samples (<10%) could not be successfully genotyped, likely due to low viral load, partial genome degradation, or suboptimal storage and transport conditions. Nevertheless, the proportion of genotyped specimens was sufficient to demonstrate a consistent predominance of HAdV-F41 across regions and years. Although molecular characterization was performed on a subset of reported positive samples, the observed genotype distribution should be interpreted as indicative of nationwide circulation patterns rather than as an exhaustive representation.

A further source of bias arises from the passive nature of the surveillance system, which depends on laboratory testing demand. The cases captured likely represent only the visible portion of the epidemiological “iceberg,” as many diarrheal episodes are not medically attended, and even fewer are subjected to etiological diagnosis. This situation is partly explained by current recommendations from international health agencies and scientific societies, which prioritize laboratory confirmation mainly for severe or hospitalized cases, or when the clinical course is unusually prolonged or complicated [30]. This underrepresentation particularly affects milder community cases and regions with limited laboratory infrastructure, as documented in other national surveillance systems [14]. In this context, the number of stool samples submitted to the national reference laboratory for genotyping reflects routine diagnostic and reporting practices rather than an active case-finding strategy, which is inherent to the design of passive surveillance systems.

Despite these limitations, this study provides a useful baseline for describing the circulation of enteric adenoviruses in Argentina within a routine, laboratory-based surveillance framework. The relatively stable detection patterns observed over the three-year period are consistent with reports from other settings and support the internal coherence of the surveillance data, while acknowledging the constraints inherent to passive reporting systems [14,15,23,25]. Accordingly, these findings should be interpreted as reflective of surveillance performance and testing practices, rather than as direct estimates of population-level incidence or prevalence.

In summary, this study offers a nationwide overview of enteric adenovirus detection and genetic diversity among children in Argentina. By integrating epidemiological and laboratory-based information, it contributes to characterizing temporal, geographic, and virological patterns under real-world surveillance conditions. Rather than implying causal shifts in disease burden, our findings highlight the importance of sustained and standardized surveillance to monitor trends, document circulating genotypes, and provide continuity for future comparative analyses. From a public health perspective, the main contribution of this work lies in documenting the outputs and operational characteristics of an established national surveillance system, including its strengths and limitations. This information provides valuable epidemiological context for ongoing monitoring of enteric adenoviruses in pediatric populations and may inform future efforts to strengthen surveillance capacity and guide research priorities on viral gastroenteritis beyond rotavirus in similar settings.

## Figures and Tables

**Table 1:** Age-specific distribution of enteric adenovirus testing and positivity in Argentina, 2022–2024. The table summarizes the number of stool samples tested, the number of adenovirus-positive notifications, positivity rates, and relative frequency proportion (RFP) across age groups and calendar years. Positivity rates represent the proportion of adenovirus-positive samples within each age group and year, while RFP indicates the proportion of samples tested in each age group relative to the total number of tests performed.

	2022				2023				2024				Total (2022–2024)			
Age	No. of Tests	HAdV (+) ^b^	%	RFP ^c^	No. of Tests	HAdV (+)	%	RFP	No. of Tests	HAdV (+)	%	RFP	No. of Tests	HAdV (+)	%	RFP
<1 y ^a^	1768	153	8.7	26.0	2238	215	9.6	29.8	2536	168	6.6	29.7	6542	536	8.2	28.7
1 year	1459	116	8.0	21.5	1713	155	9.0	22.8	1974	124	6.3	23.1	5146	395	7.7	22.5
2–4 y	2104	122	5.8	31.0	2080	132	6.3	27.7	2180	108	5.0	25.6	6364	362	5.7	27.9
5–9 y	1457	73	5.0	21.5	1480	86	5.8	19.7	1837	78	4.2	21.5	4774	237	5.0	20.9
Total	6788	464	6.8	100	7511	588	7.8	100	8527	478	5.6	100	22,826	1530	6.7	100

^a^ y: year/s. ^b^ (+): positive samples. ^c^ RFP: relative frequency proportion of samples tested.

**Table 2:** Distribution of enteric adenovirus testing and positivity by healthcare setting in Argentina, 2022–2024. The table summarizes the number of stool samples tested, the number of adenovirus-positive notifications, positivity rates, and the relative frequency proportion (RFP) according to healthcare setting (outpatient/emergency department and inpatient) and calendar year. Positivity rates represent the proportion of adenovirus-positive samples within each clinical setting and year, while RFP indicates the proportion of samples tested in each setting relative to the total number of tests performed.

	2022				2023				2024				Total (2022–2024)			
Setting	No. of Tests	HAdV (+) ^b^	%	RFP ^c^	No. of Tests	HAdV (+)	%	RFP	No. of Tests	HAdV (+)	%	RFP	No. of Tests	HAdV (+)	%	RFP
Outpatient/ED ^a^	2478	273	11.0	36.5	2856	292	10.2	38.0	2976	239	8.0	34.9	8310	804	9.7	36.4
Inpatient	4310	191	4.4	63.5	4655	296	6.4	62.0	5551	239	4.3	65.1	14,516	726	5.0	63.6
Total	6788	464	6.8	100	7511	588	7.8	100	8527	478	5.6	100	22,826	1530	6.7	100

^a^ ED: Emergency Department. ^b^ (+): positive samples. ^c^ RFP: relative frequency proportion of samples tested.

**Table 3:** Distribution of enteric adenovirus testing and positivity by geographic region in Argentina, 2022–2024. The table summarizes the number of stool samples tested, the number of adenovirus-positive notifications, positivity rates, and the relative frequency proportion (RFP) across geographic regions and calendar years. Positivity rates represent the proportion of adenovirus-positive samples within each region and year, while RFP indicates the proportion of samples tested in each region relative to the total number of tests performed.

	2022				2023				2024				Total (2022–2024)			
Region ^a^	No. of Tests	HAdV (+) ^b^	%	RFP ^c^	No. of Tests	HAdV (+)	%	RFP	No. of Tests	HAdV (+)	%	RFP	No. of Tests	HAdV (+)	%	RFP
North	1255	40	3.2	18.5	1331	129	9.7	17.7	1424	51	3.6	16.7	4010	220	5.5	17.6
Center	4974	346	7.0	73.3	5498	351	6.4	73.2	6443	369	5.7	75.6	16,915	1066	6.3	74.1
South	559	78	14.0	8.2	682	108	15.8	9.1	660	58	8.8	7.7	1901	244	12.8	8.3
Total	6788	464	6.8	100	7511	588	7.8	100	8527	478	5.6	100	22,826	1530	6.7	100

^a^ North region includes the provinces of Catamarca, Chaco, Corrientes, Formosa, Jujuy, La Rioja, Misiones, Salta, Santiago del Estero, and Tucumán. Center region includes the provinces of Buenos Aires, Córdoba, Entre Ríos, La Pampa, Mendoza, San Juan, San Luis, Santa Fe, and the Autonomous City of Buenos Aires. South region includes the provinces of Chubut, Neuquén, Río Negro, Santa Cruz, and Tierra del Fuego, Antártida e Islas del Atlántico Sur. ^b^ (+): positive samples. ^c^ RFP: relative frequency proportion of samples tested.

## Data Availability

The data supporting the findings of this study are not publicly available due to data protection and confidentiality restrictions, as they were generated within the framework of the national epidemiological surveillance system in Argentina. Requests for access to aggregated data may be submitted to the Area of Surveillance of the National Directorate of Epidemiology and Strategic Health Information, Ministry of Health of Argentina (arevigilanciamsal@gmail.com), and are subject to institutional authorization and compliance with applicable regulatory and ethical requirements.

## Abbreviations

The following abbreviations are used in this manuscript:

HAdV	Human adenovirus
SNVS2.0	National Health Surveillance System
EW	Epidemiological week
NT	Non-typeable
